# Molecular Dynamics Simulation as a Tool to Identify Mutual Synergistic Folding Proteins

**DOI:** 10.3390/ijms24021790

**Published:** 2023-01-16

**Authors:** Csaba Magyar, Bálint Zoltán Németh, Miklós Cserző, István Simon

**Affiliations:** 1 Institute of Enzymology, Research Centre for Natural Sciences, Eötvös Loránd Research Network, 1117 Budapest, Hungary; 2 Department of Physiology, Faculty of Medicine, Semmelweis University, 1094 Budapest, Hungary

**Keywords:** mutual synergistic folding, disordered proteins, oligomeric proteins, molecular dynamics simulations, cooperative two-state unfolding

## Abstract

Mutual synergistic folding (MSF) proteins belong to a recently emerged subclass of disordered proteins, which are disordered in their monomeric forms but become ordered in their oligomeric forms. They can be identified by experimental methods following their unfolding, which happens in a single-step cooperative process, without the presence of stable monomeric intermediates. Only a limited number of experimentally validated MSF proteins are accessible. The amino acid composition of MSF proteins shows high similarity to globular ordered proteins, rather than to disordered ones. However, they have some special structural features, which makes it possible to distinguish them from globular proteins. Even in the possession of their oligomeric three-dimensional structure, classification can only be performed based on unfolding experiments, which are frequently absent. In this work, we demonstrate a simple protocol using molecular dynamics simulations, which is able to indicate that a protein structure belongs to the MSF subclass. The presumption of the known atomic resolution quaternary structure is an obvious limitation of the method, and because of its high computational time requirements, it is not suitable for screening large databases; still, it is a valuable in silico tool for identification of MSF proteins.

## 1. Introduction

Mutual synergistic folding (MSF) proteins belong to a recently emerged subclass of disordered proteins, which are ordered in their oligomeric state, but disordered in their monomeric state [1,2]. For ordering the structure of a traditional disordered protein, a template with an already stable, ordered structure is needed. In the case of MSF proteins, folding happens cooperatively with the association of the disordered subunits. The structural organization of these proteins is highly dynamic, depending on their oligomeric state, probably resulting from their local concentrations. There have been several breakthroughs in the history of protein structure research, starting with the atomic resolution structure determination of globular proteins [3], through the discovery of transmembrane protein structures [4], and the “coupled folding and binding” mechanisms of disordered proteins [5,6]. With the discovery of these new protein classes, the original concept was also broken, and the knowledge of a protein’s three-dimensional structure provides us with relevant information. In the case of transmembrane proteins, information about the position of the membrane is not included in the structures, thus novel methods were needed for their localization [7]. For disordered proteins, the structure of a single ordered–disordered complex does not tell us anything about the probably different structures of other possible complexes, which can be formed with different interaction partners [5]. These newly discovered protein classes had one property in common, their amino acid composition showed a significant difference from the previously known ones. The recognition of MSF proteins as a novel protein subclass did not bring such a breakthrough; furthermore, their amino acid composition does not differ substantially from that of globular proteins [8,9,10,11]. It is still unclear why MSF proteins evolved in such a manner. What kind of advantage can be resulting from the unfolding of the whole protein upon losing its native oligomeric structure? There is an ongoing research project unraveling the role of this potential concentration-dependent regulation mechanism and an investigation of the structural organization of MSF proteins.

There is only a single published collection of experimentally validated MSF proteins available, the Mutual Folding Induced by Binding (MFIB) database [2]. This database contains 205 proteins in several different oligomeric assemblies, homodimers being the most populated assembly class containing 98 protein entries. Among these 98 entries, 31 structures belong to the coils and zyppers structural class, which have special structural features, and thus should not be handled together with traditional globular proteins. Although the small size of the database made it difficult to understand the structural features behind this phenomenon, we recently managed to identify some special structural features on the largest homo- and hetero-dimeric subsets of the MFIB database [8,9,10]. We are currently working on the development of a structure-based prediction method, which could be used to increase the number of identified MSF proteins. Since experimental validation is needed for a reliable classification of MSF proteins during the construction of the MFIB database, a large number of research papers had to be processed to filter out reliable experimental evidence. Chances are high that there are a lot more oligomeric MSF protein structures present in the Protein Data Bank (PDB) database, which could not be classified as MSF proteins because of the lack of unfolding experiments. Since a larger database of MSF proteins would facilitate the ongoing research projects, the need for a tool has emerged, which could be used in the absence of unfolding experimental results to support the MSF classification. In this work, we propose a simple protocol based on molecular dynamics (MD) simulations, performed on the experimental oligomeric and hypothetical monomeric structures of proteins.

## 2. Results

Molecular dynamics simulations are known to reproduce well the thermal unfolding of proteins [12]. The basic idea was to compare the behavior of the hypothetical monomeric and experimental dimeric structures of MSF and globular proteins using all-atom NPT MD simulations. Since many MFIB entries are human proteins, simulations were performed at 310 K. This facilitates slightly larger thermal fluctuations compared to a 300 K simulation and prevents undesired thermal denaturation at the same time.

**Figure 1:**
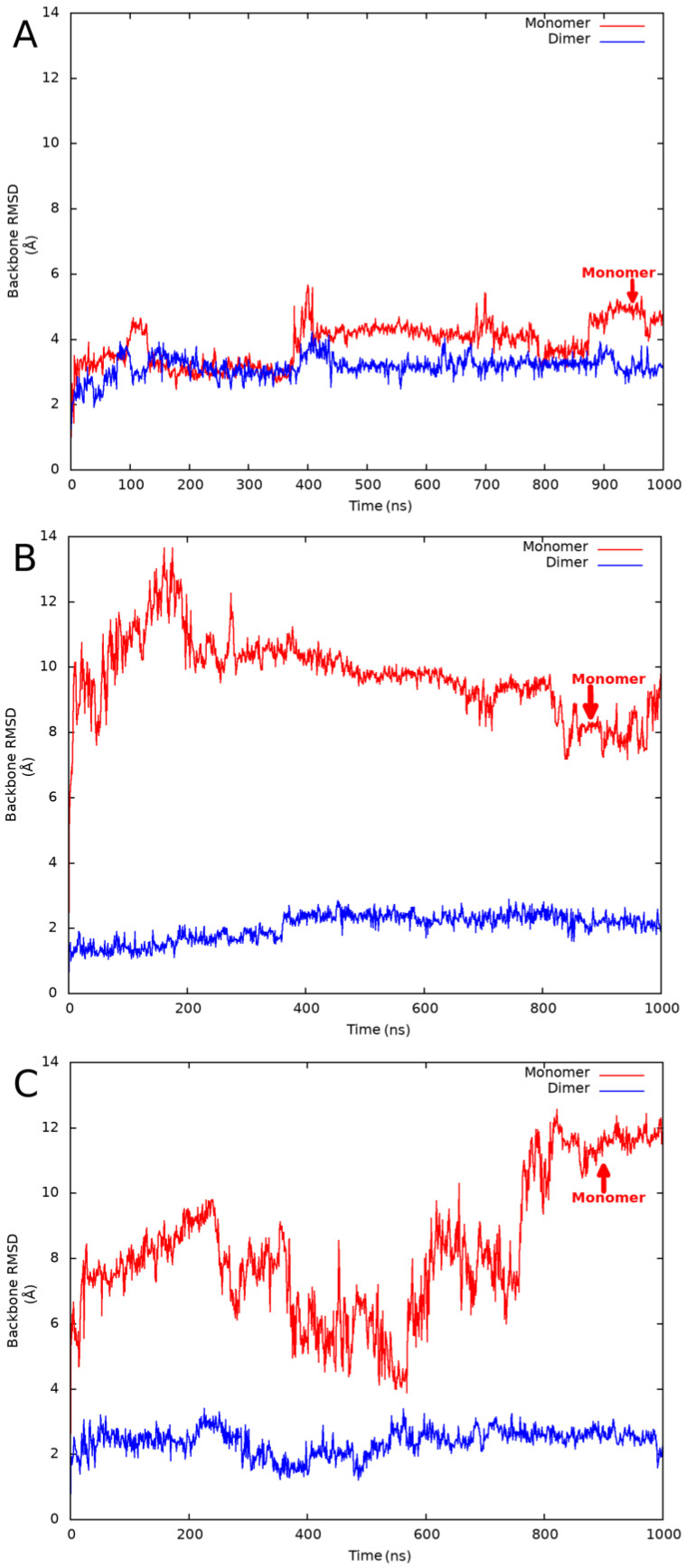
Example backbone RMSD plots obtained from the monomeric (red curve) and dimeric (blue curve) MD simulation of the (**A**) 4low globular protein; (**B**) 4cn0 expected MSF protein; and (**C**) 4me7 MSF protein.

Since we planned the investigation of several proteins, we had to find the optimal simulation strategy, which provides simulation trajectories close to a quasi-equilibrium state but is computationally efficient at the same time. During the initial phase of the protocol development, we experimented with different (250, 500, 1000, and 4 × 250 ns) simulation times and compared the results obtained on a single globular and MSF test protein. We found that a single 1000 ns simulation provided the most converged backbone root-mean-square deviation (RMSD) plots and the highest average RMSD values among all simulations. Only backbone atoms were taken into account during the RMSD calculations, and the structure obtained after the initial default relaxation protocol was used as a reference. See Figure 1 for example backbone RMSD plots obtained for a globular, a validated MSF, and an expected MSF protein. 

The size of the fully prepared systems was in the range of 10–28 k atoms. The longest simulations run almost 3 days long on our Nvidia Quadro P6000 GPU card, with a typical GPU runtime of about 4 days per protein including both dimeric and monomeric simulations. For the last couple of simulations, GPU time on an Nvidia RTX A4500 card was also used to perform the MD simulations. Even with the doubled hardware resource, performing longer simulations was not realistic in a reasonable time.

In this work, we investigate only homodimers as a model system to understand the structural background of MSF proteins. These are the simplest oligomeric structures built up from two identical protein chains. Higher oligomeric forms, such as tetra or hexamers, could exist in several oligomeric forms (as dimers for example), which would complicate the problem. Since the mostly populated MFIB assembly class is the homodimeric one, this approach seems to be adequate. We used homodimeric structures from the MFIB database, and entries from our reference globular homodimeric (GHOD) dataset presented in our previous publication [10]. The usage of homodimers also simplified the simulation protocol, because only one monomeric simulation had to be performed. Already on heterodimeric structures, additional monomeric simulations of the second proteins chain should have been performed. Because of the high computational time required for a 1000 ns simulation, screening of the complete MFIB and GHOD datasets using our own hardware resources was not realistic; thus, we selected six MSF and six globular proteins. We expect that there are several MSF proteins in our globular GHOD dataset, which were not identified as MSF proteins because of lacking experimental evidence. During the selection of the globular proteins, we were applying a dual strategy. On the one hand, we tried to avoid the selection of possible MSF proteins as real globular references. On the other hand, we were looking for suspicious proteins expected to belong to the MSF subclass, as an internal test set. Real globular proteins were selected among GHOD entries with low buried/accessible peptide bond ratios and low Shannon information entropy values, which were found typical for globular proteins in our previous publication [10]. We also selected six suspicious proteins from the GHOD dataset with high buried/accessible peptide bond ratios and high Shannon information entropy values, which were found to be characteristic of MSF proteins. The simulation of these 18 proteins took more than 2 months of GPU time. 

We performed 1000 ns all-atom NPT MD simulations at 310 K temperature on both the dimeric and a hypothetical monomeric structures using the Desmond [13] program of the Schrödinger 2022-3 [14] software package, with the Optimized Potentials for Liquid Simulations (OPLS) forcefield [15]. We were analyzing the last 50 ns of the simulations by calculating average backbone RMSD values using the initially relaxed structure as a reference. The results are listed in Table 1. We have found that the RMSD values obtained from the monomeric simulations were higher in every case. In the case of the real globular homodimers, the ratio of the monomeric/dimeric average RMSD values was in the range of 1.20–1.62. For the validated MSF and expected MSF proteins, the ratio was in the range of 2.24–5.33 and 2.26–4.59, respectively. This behavior was expected because MSF proteins should not be stable in their theoretical monomeric state. Unfolding may not happen during 1000 ns simulations, but the significantly higher RMSD values support our hypothesis. Among the globular proteins, the highest average RMSD values were obtained for a putative XRE family transcriptional regulator protein (PDB code: 2ofy). Despite the large absolute values, the monomeric/dimeric RMSD ratio was only 1.36, which seems to be a typical value for globular proteins. For every validated or expected MSF protein, the ratio was at least 2.24. Among the MSF proteins, the lowest monomeric RMSD value was observed for the *E. coli* Met repressor (PDB code: 1cmb) protein. This relatively low RMSD value was accompanied by the lowest dimeric RMSD value in this group, resulting in a high ratio of 2.51. From these observations, we concluded that, independent from the absolute RMSD values, high ratios of the monomeric/dimeric RMSD values are characteristic of MSF protein structures. Because ratios obtained for globular and MSF proteins are well separated, a threshold value close to the lowest observed ratio for experimentally validated MSF proteins can be used to identify MSF proteins. Based on the simulation already performed, we suggest a threshold value of 2.2 for the differentiation between MSF and globular proteins. This threshold can be fine-tuned by future simulations on MSF and globular proteins. 

A structured-based prediction method is currently in development to identify MSF proteins based on their atomic resolution quaternary structure. The method will include the identification of buried/accessible residues using three-dimensional structures and Shannon information entropies calculated from amino acid sequences. The presented protocol is indispensable for the optimization of the parameters in our prediction method. This protocol is the only tool for validation of the initial predictions in the absence of experimental unfolding experiments.

## 3. Discussion

Molecular dynamics simulation is a powerful tool for investigating protein unfolding. The following example demonstrates well the effectiveness of MD simulations. We uncovered an entry in our GHOD database (PDB code 1wv9), which was erroneously classified as a globular homodimer. We obtained 1.70 Å and 1.69 Å RMSD values for the monomeric, and dimeric simulations, respectively. We checked the simulation trajectory, which showed that the two protein chains were separated during the simulation. We checked the PDBePISA [16] quaternary structure prediction of this entry, and it proved to be a monomeric structure with two protein chains in the asymmetric crystallographic unit. 

Both validated and predicted MSF proteins show significantly larger fluctuations in their monomeric form, resulting in higher monomeric/dimeric average RMSD ratios. We are confident that proteins with a monomeric/dimeric RMSD ratio over 2.2 can be considered MSF proteins. This protocol can be applied to expected MSF proteins, identified by our development structure-based prediction method. The experiment-based MFIB database can be expanded by the MD simulation validated predictions, resulting in a significantly larger dataset. This new larger dataset is planned to be used for the development of a sequence-based MSF prediction method. In our recent work [10], we already showed that Shannon information entropy values calculated from the amino acid sequences are useful measures for the differentiation of MSF and globular proteins. The increased database size could provide an improved sequence-based prediction method in terms of statistical significance. With the recent development of the highly accurate AlphaFold-Multimer [17] oligomeric protein structure prediction method, we will even have the possibility to model the structure of proteins identified by the sequence-based prediction method. The current protocol can be possibly improved for use on Alphafold-Multimer provided model structures, possibly requiring a stable simulation trajectory and adequately low average RMSD values obtained from MD simulation performed on the supposedly stable oligomeric structures. 

## 4. Materials and Methods

MSF protein structures were downloaded from the MFIB [2] database, using the modified structures where available. Globular protein structures were downloaded from the PDB database [18]. The structures were prepared for MD simulations using the Protein Preparation module [19] of the Schrödinger software package [14] using default options with the addition of the fill-in missing side-chains + loops, and cap termini extra options. Ligands were deleted from the prepared structures. After the hydrogen bond optimization step, a hydrogen-only minimization and a subsequent all-atom minimization step was performed using 0.7 Å RMSD termination criteria. The default 0.3 Å value resulted in an unstable monomeric simulation already at the beginning of the default relaxation protocol. The resulting dimeric structures were saved for the MD simulations. A hypothetical monomeric structure was created by deleting the second protein chain from the already prepared dimeric structures.

The Desmond [13] program was used for the all-atom NPT MD simulations performed at 310 K temperature and 1.01325 bar pressure using the default parameters (2 fs time step, 9 Å Coulombic cutoff, Nose-Hoover chain thermostat [20], and Martyna-Tobias-Klein isotropic barostat [21]), with the OPLS4 forcefield [15]. During NPT simulations, the number of atoms (N), the pressure (P), and the temperature (T) of the system are kept constant (isothermal–isobaric ensemble). The systems were set using the System Builder module of the Schrödinger Suite 2022-3 [14] software package. The simple point charge (SPC) [22] solvent model was used for setting up the simulation systems. In the case of the dimeric structures, a distance of 10 Å was used to set up a triclinic periodic boundary box. In the case of the monomeric structures, because of the higher expected fluctuations, a moderately increased distance of 12 Å was used to minimize artificial contacts between protein chains in neighboring simulation boxes. Since the number of atoms, and consequently the simulation time depends heavily on this distance, no larger distances were allowed. Finally, the systems were neutralized by the addition of the appropriate number of sodium or chloride ions. 

After applying the default relaxation protocol 1000 ns, NPT MD simulations were performed at 310 K temperature, and during the simulations, 1000 frames were saved. After the simulations, the backbone RMSD vs. time plots were visually checked if the simulation produced a convergent trajectory. For the analysis, the last 50 frames were used to calculate an average backbone RMSD value with the run analyse_simulation.py program provided by the Schrödinger 2022-3 software package [14], using the structure obtained after the initial default relaxation protocol as reference (frame 0).

For the selection of the real globular and expected MSF proteins, we used the methodology described in our previous publication [10]. The buried/accessible peptide bond ratios were calculated using relative solvent accessible surface values calculated by the FreeSASA 2.03 [23] program. The Shannon information entropy values [24] were calculated using our own programs using BioPython [25], utilizing the ELKH Cloud hardware infrastructure [26].

## 5. Conclusions

The presented protocol can be used to validate the MSF classification of oligomeric proteins in possession of their atomic resolution quaternary structures. The protocol can be used to remove the hindrance originating from the limited size of the MFIB database containing only experimentally validated MSF proteins. Unfortunately, the protocol is not appropriate to screen the whole PDB database using commodity hardware resources, but it can be used to validate proteins identified by a structure-based MSF prediction method. Hereby, the size of the MSF database can be increased significantly, which will serve as a good basis for future work on the characterization and sequence-based prediction of MSF proteins. 

## Figures and Tables

**Table 1:** Average RMSD values obtained from the monomeric and dimeric MD simulations.

PDB Code	Structure Class	Monomeric RMSD [Å]	Dimeric RMSD [Å]	Ratio
1m2d	globular	3.59	3.00	1.20
1oqj	globular	5.06	4.23	1.20
2ofy	globular	5.94	4.37	1.36
3n4w	globular	1.85	1.43	1.29
4low	globular	4.47	3.12	1.43
4rd7	globular	4.48	2.76	1.62
1gyx	globular *	7.15	3.17	2.26
2ge7	globular *	12.13	2.64	4.59
3n8b	globular *	10.49	2.39	4.39
4aeq	globular *	8.64	3.10	2.79
4cn0	globular *	8.50	2.13	3.99
5fs4	globular *	8.41	3.57	2.36
1cmb	MSF	5.18	2.06	2.51
2ay0	MSF	7.14	2.58	2.77
2cpg	MSF	6.63	2.91	2.28
3wpd	MSF	11.77	2.21	5.33
4ec7	MSF	5.70	2.54	2.24
4me7	MSF	10.13	2.63	3.85

* Expected MSF.

## Data Availability

Not applicable.
